# The expression of *Ldh-c* in the skeletal muscle of plateau pika (*Ochotona curzoniae*) enhances adaptation to a hypoxic environment

**DOI:** 10.1242/bio.024943

**Published:** 2017-09-15

**Authors:** Zhi F. An, Deng B. Wei, Lian Wei, Yang Wang, Lin N. Wei

**Affiliations:** 1 State Key Laboratory of Plateau Ecology and Agriculture, Qinghai University 10743, Xining, Qinghai 810016, China; 2 Research Center for High Altitude Medicine, Qinghai University, Xining, Qinghai 810016, China; 3 College of Eco-Environmental Engineering, Qinghai University, Xining, Qinghai 810016, China

**Keywords:** *Ldh-c*, Plateau pika (*Ochotona curzoniae*), Hypoxia, Qinghai-Tibet Plateau, Skeletal muscle

## Abstract

The plateau pika (*Ochotona curzoniae*) is a species of sprint-running alpine animals in the Qinghai-Tibet Plateau, which is a harsh highland hypoxic environment. *Ldh-c* is expressed in the testis, sperm and somatic tissues of plateau pika. To reveal the role and physiological mechanisms of sperm-specific lactate dehydrogenase (LDH-C_4_), in plateau pika to adapt to hypoxic environment, an adenoviral line of pMultiRNAi-*Ldhc* was constructed and injected into the bilateral biceps femoris of the hind legs. The swimming times of the pikas, and the *Ldh-c* expression levels, total LDH activities and ATP levels in skeletal muscle, were measured after the pikas were raised in the trapped site for 5 days. Our results showed that after *Ldh-c* was silenced, the sprint-running ability (swimming time) of the plateau pikas was significant decreased, and the total LDH activities and ATP levels were reduced by 28.21% and 27.88%, respectively. Our results indicated that expression of *Ldh-c* in the skeletal muscle of plateau pika increased anaerobic glycolysis and enhanced adaptation to highland hypoxic environments.

## Abstract (precis)

**Summary:** The expression of *Ldh-c* in the skeletal muscle of plateau pika (*Ochotona curzoniae*) increases anaerobic glycolysis, resulting in enhanced sprint-running capacity and adaptation to a hypoxic environment.

## INTRODUCTION

The plateau pika (*Ochotona curzoniae*) is an endemic species in the Qinghai-Tibet Plateau and iinhabits meadows at an altitude of 3000-5000 m. Hypoxia is an obvious abiotic stressor in high Tibetan plateau, and pikas can significantly affect ecological diversity in the Qinghai-Tibet Plateau (Smith and Foggin, 1999; Lai and Smith, 2003). Over long-term evolution, the pika underwent a series of strategies for adaptation to the hypoxic environment physiologically and genomically (He et al., 1994; Wei et al., 2006; [Bibr BIO024943C22][Bibr BIO024943C23]; Zhu et al., 2009; Sun et al., 2013). Recently, we reported that the testis-specific lactate dehydrogenase gene (*Ldh-c*), regarded originally to be expressed in the testis and spermatozoa, was detected in the somatic tissues of plateau pika (Wang et al., 2013). Using a specific inhibitor (*N*-isopropyl oxamate) of sperm-specific lactate dehydrogenase (LDH-C_4_), we demonstrated that LDH-C_4_ enhanced the pikas’ sprint-running capacity, which has an important role in adaption to highland hypoxic environment in plateau pika (Wang et al., 2015).

LDH-C_4_ is expressed in the spermatozoa and testis of birds and mammals (Goldberg, 1964, 1975, 1984; Coonrod et al., 2006), and is one of the LDH isoenzymes (Blanco and Zinkham, 1963; Goldberg, 1963). LDH-C_4_ has higher affinity for pyruvate than lactate, and catalyses the conversion of pyruvate to lactate in the semen of humans and other species (Clausen and Ovlisen, 1965; Wilkinson and Withycombe, 1965; Coronel et al., 1983; LeVan and Goldberg, 1991; Wong et al., 1997; Hereng et al., 2011; Wang et al., 2016), which is crucial for glycolysis to continue production of adenosine triphosphate (ATP) (Coronel et al., 1983; LeVan and Goldberg, 1991; Odet et al., 2011). LDH-C_4_, is the key factor of sperm glycolysis, which has an important role in sperm motility to provide ATP (Coronel et al., 1983; Williams and Ford, 2001; Mukai and Okuno, 2004; Odet et al., 2011). In our previous studies, we found that the affinity of LDH-C_4_ for pyruvate was ∼90-fold higher than that for lactate, and it was not easily inhibited by high lactate concentration (Wang et al., 2016). LDH-C_4_ was conducive to catalysis of the conversion of pyruvate to lactate. When *N*-isopropyl oxamate was injected into the skeletal muscle of plateau pika, the total LDH activities, lactate content and ATP levels were reduced by 37.12%, 66.27% and 32.42%, respectively, and antifatigue ability was also decreased significantly (Wang et al., 2015). These results indicated that *Ldh-c* expressed in the skeletal muscle of plateau pika is conducive to catalysis of the conversion of pyruvate to lactate, and accelerates anaerobic glycolysis to generate ATP.

However, the specific LDH-C_4_, inhibitor (*N*-isopropyl oxamate) had a strong and specific effect on LDH-C_4_, and it still had some inhibitory effects on LDH-A_4_ and LDH-B_4_ (Wang et al., 2015). Therefore, in the current study, to further reveal the effect of *Ldh-c* on the performance tolerance of plateau pika, we investigated the effect of *Ldh-c* expression levels on pika sprint-running capacity by silencing *Ldh-c* in skeletal muscle with the method of RNAi. The molecular mechanism was elucidated by measuring LDH activity, lactate content and ATP level in pika skeletal muscle.

## RESULTS

### Swimming time

**Fig. 1.**
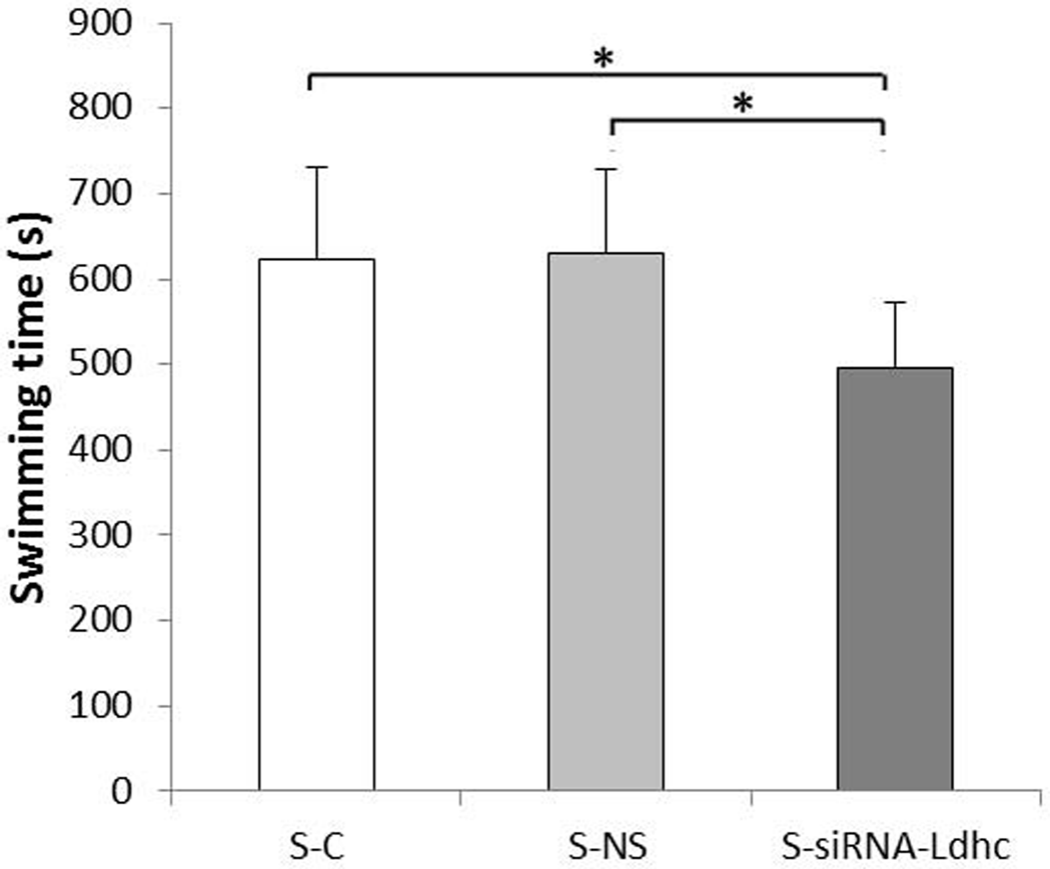
**Swimming time of plateau pika in the different treatment groups.** The swimming times of the C, NS and siRNA-*Ldhc* groups were 633±128 s, 643±140 s and 476±92 s, respectively. *P<0.05. Water temperature, 9-10°C. The sample size was eight for each group.

We examined the swimming time of plateau pika with different treatments to identify the effect of siRNA-*Ldhc* on performance tolerance. Statistical results showed that it was significantly higher in the control (C) group (633±140 s) and nonspecific sequence group (NS) group (643±128 s) compared with the siRNA-*Ldhc* group (476±92 s) (*P*<0.05) (Fig. 1).

### qRT-PCR analysis of *Ldh-a*, *Ldh-b* and *Ldh-c* mRNA expression

**Fig. 2.**
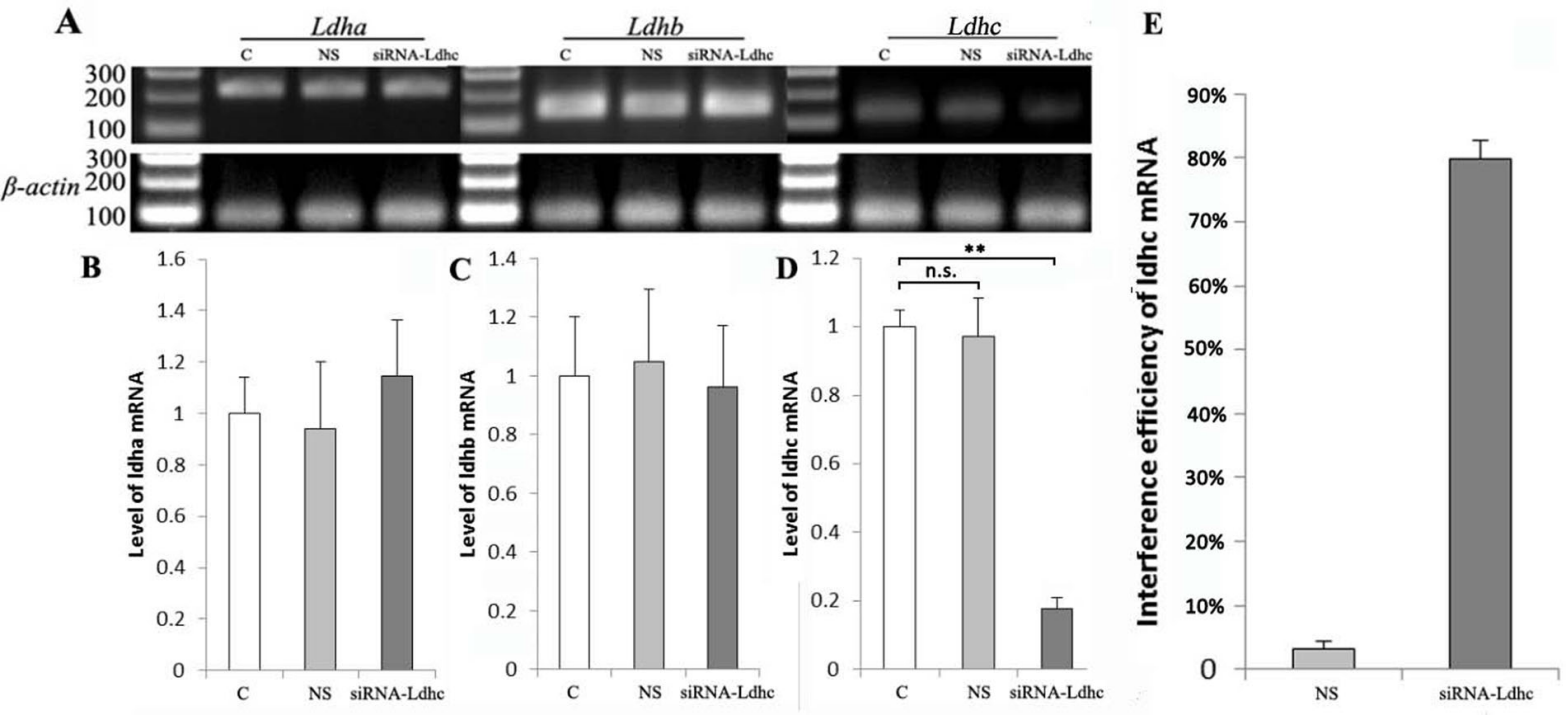
**Quantification of *Ldh-a*, *Ldh-b* and *Ldh-c* mRNA levels in plateau pika skeletal muscle of the different treatment groups.** (A) The electrophoresis results of real-time PCR for *Ldh-a*, *Ldh-b*, *Ldh-c* and *β-actin* in plateau pika skeletal muscle. (B) Quantification of *Ldh-a* mRNA expression levels in plateau pika skeletal muscle. The C, NS and siRNA-*Ldhc* group results were 1.00±0.11, 0.94±0.28 and 1.15±0.19, respectively. (C) Quantification of *Ldh-b* mRNA expression levels in plateau pika skeletal muscle. The C, NS and siRNA-*Ldhc* group results were 1.00±0.19, 1.04±0.24 and 0.95±0.21, respectively. (D) Quantification of *Ldh-c* mRNA expression levels in plateau pika skeletal muscle. The C, NS and siRNA-*Ldhc* group results were 1.00±0.05, 0.97±0.12 and 0.18±0.04, respectively. (E) Interference efficiency of *Ldh-c* after RNAi. The interference efficiency of the NS group and siRNA-*Ldhc* group was 2.80±0.45% and 82.18±3.52%, respectively. **P<0.01; n.s., not significant. The sample size was eight for each group.

Since *Ldh-c* has high homology with *Ldh-a* and *Ldh-b*, we examined *Ldh-a*, *Ldh-b* and *Ldh-c* mRNA levels using qRT-PCR assays to confirm whether *Ldh-a* and *Ldh-b* were interfered with by siRNA-*Ldhc* and the adenovirus simultaneously (Fig. 2A). In the pika skeletal muscle of the C, NS and siRNA-*Ldhc* groups, *Ldh-a* mRNA levels were 1.00±0.11, 0.94±0.28 and 1.15±0.19, respectively; *Ldh-b* mRNA levels were 1.00±0.19, 1.04±0.24 and 0.95±0.21, respectively; and *Ldh-c* mRNA levels were 1.00±0.05, 0.97±0.12 and 0.18±0.04, respectively (Fig. 2B,C,D). There was no statistically significant difference in the expression levels of *Ldh-a* and *Ldh-b* mRNA among the three groups. Compared to the C group, the interference efficiency of *Ldh-c* in the siRNA-*Ldhc* group was 82.18% in the skeletal muscle, while that of the NS group was only 2.80% (Fig. 2E).

### Western blot analysis of LDHC protein expression

**Fig. 3.**
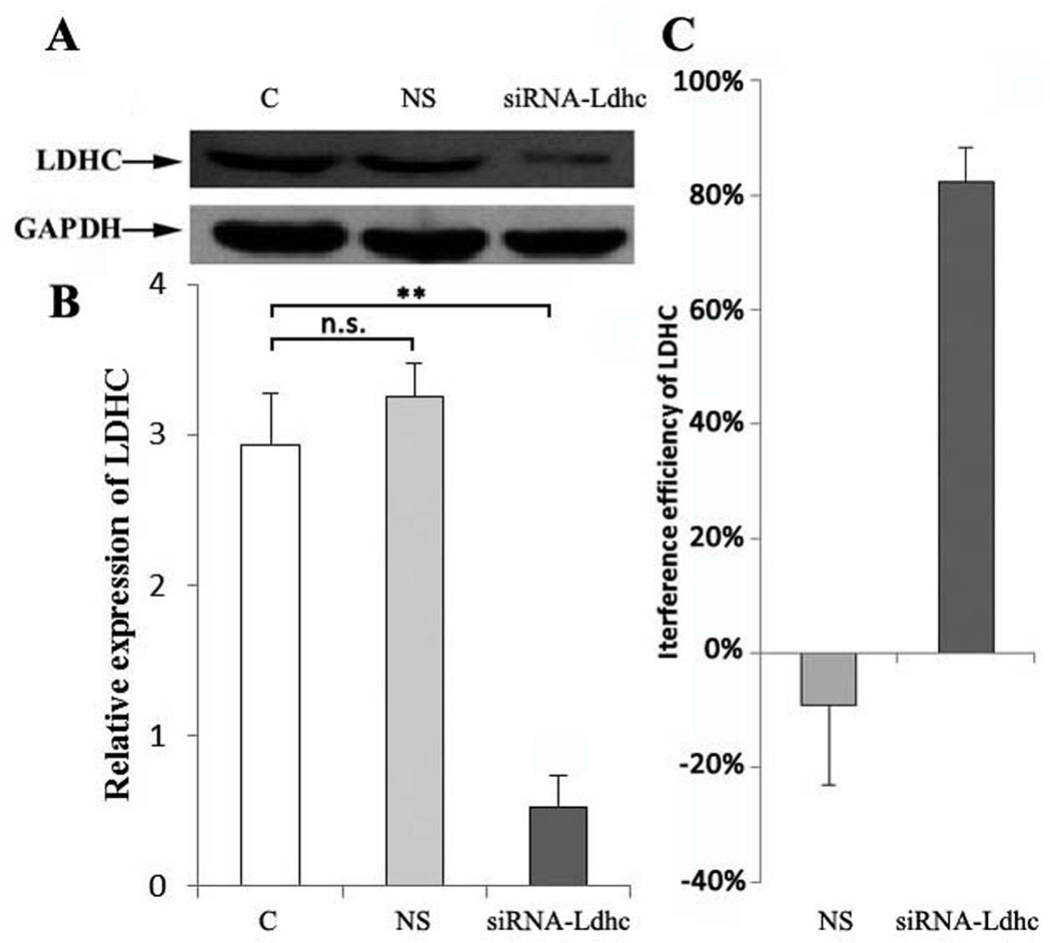
**Quantification of LDHC levels in plateau pika skeletal muscle of the different treatment groups.** (A) Western blot analysis of LDHC and GAPDH protein in plateau pika skeletal muscle. (B) Quantification of LDHC protein expression levels in plateau pika skeletal muscle. The C, NS and siRNA-*Ldhc* group results were 2.93±0.34, 3.25±0.22 and 0.53±0.21, respectively. (C) The interference efficiency of siRNA on LDHC expression in the NS group and siRNA-*Ldhc* group was –6.99±13.83% and 82.29±9.20%, respectively, compared to the C group. **P<0.01; n.s., not significant. The sample was eight for each group.

The protein level of LDHC was examined by western blotting in plateau pika skeletal muscle. The relative expression levels of LDHC protein in the C, NS and siRNA-*Ldhc* groups were 2.93±0.34, 3.24±0.22 and 0.53±0.21, respectively. Statistical results showed that LDHC protein expression was significantly higher in the C group and NS group than in the siRNA-*Ldhc* group. There was no significant difference in LDHC protein expression between the C and NS groups (*P*>0.05) (Fig. 3A,B). LDHC expression levels in the NS group and siRNA-*Ldhc* group decreased by −6.99±13.83% and 82.29±9.20%, respectively, compared to the C group (Fig. 3C).

### LDH activity, LD content and ATP level in plateau pika tissues

**Fig. 4.**
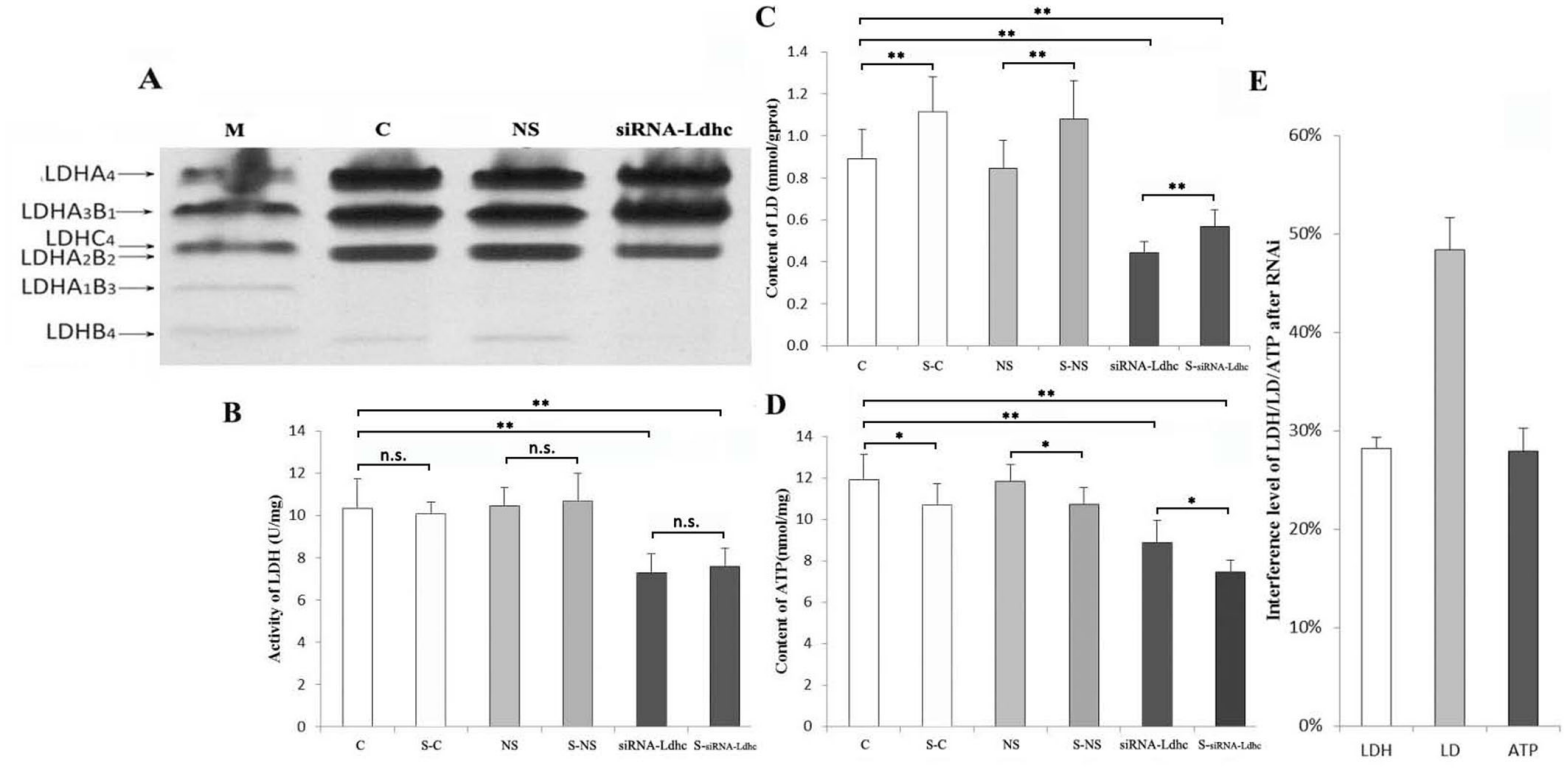
**LDH isozyme electrophoresis, LDH activities, LD content and ATP levels in pika skeletal muscle.** (A) LDH isozyme electrophoresis. Lanes C, NS and siRNA-*Ldhc* represent the skeletal muscle in the groups; M, marker (the serum of pikas). (B) LDH activities of the C, S-C, NS, S-NS, siRNA-*Ldhc* and S-siRNA-*Ldhc* groups were 10.333±1.389 U/mg, 10.049±0.582 U/mg, 10.430±0.869 U/mg, 10.656±1.319 U/mg, 7.269±0.931 U/mg and 7.584±0.861 U/mg, respectively. (C) LD content of the C, S-C, NS, S-NS, siRNA-*Ldhc* and S-siRNA-*Ldhc* groups was 0.89±0.14 mmol/g, 1.12±0.17 mmol/g, 0.84±0.13 mmol/g, 1.08±0.18 mmol/g, 0.44±0.06 mmol/g and 0.57±0.08 mmol/g, respectively. (D) ATP levels of the C, S-C, NS, S-NS, siRNA-*Ldhc* and S-siRNA-*Ldhc* groups were 11.909±1.231 nmol/mg, 10.691±1.033 nmol/mg, 11.854±0.807 nmol/mg, 10.716±0.801 nmol/mg, 8.868±1.066 nmol/mg and 7.459±0.566 nmol/mg, respectively. (E) The interference levels of siRNA on LDH activity, LD content and ATP level were 28.21±1.10%, 48.38±3.29% and 27.88±2.33%, respectively. *P<0.05; **P<0.01; n.s., not significant. The sample size was eight for each group.

As shown in Fig. 4A, LDH isozyme electrophoresis results revealed that the main LDH isoenzymes found in the skeletal muscle of plateau pikas were LDH-A_4_, LDH-A_3_B, LDH-C_4_, LDH-A_2_B_2_ and LDH-B_4_; in the siRNA-*Ldhc* group, the LDH-C_4_ content was obviously downregulated compared to the C group and NS group.

LDH activities, lactic acid (LD) content and ATP levels in pika skeletal muscle decreased after injecting the adenovirus of pMultiRNAi-*Ldhc*. As shown in Fig. 4B,C,D, LDH activities of the C, swimming control (S-C), NS, swimming nonspecific sequence (S-NS), siRNA-*Ldh* and S-siRNA-*Ldhc* groups were 10.333±1.389 U/mg, 10.049±0.582 U/mg, 10.43±0.869 U/mg, 10.656±1.319 U/mg, 7.269±0.931 U/mg and 7.584±0.861 U/mg, respectively; the LD content of the C, S-C, NS, S-NS, siRNA-*Ldhc* and S-siRNA-*Ldhc* groups were 0.89±0.14 mmol/g, 1.12±0.17 mmol/g, 0.84±0.13 mmol/g, 1.08±0.18 mmol/g, 0.44±0.06 mmol/g and 0.57±0.08 mmol/g, respectively; the ATP levels of the C, S-C, NS, S-NS, siRNA-*Ldhc* and S-siRNA-*Ldhc* groups were 11.909±1.231 nmol/mg, 10.691±1.033 nmol/mg, 11.854±0.807 nmol/mg, 10.716±0.801 nmol/mg, 8.868±1.066 nmol/mg and 7.459±0.566 nmol/mg, respectively. LDH activities were significantly lower in the siRNA-*Ldhc* and S-siRNA-*Ldhc* groups compared with the C, S-C, NS and S-NS groups (*P*<0.01), while there was no significant difference between the LDH activities of the siRNA-*Ldhc* group and S-siRNA-*Ldhc* group (*P*>0.05) (Fig. 4B). LD content was significantly lower in the siRNA-*Ldhc* group and S-siRNA-*Ldhc* group compared with the C, S-C, NS and S-NS groups (*P*<0.01); there was a highly significant difference between the resting and swimming states of the plateau pika (C versus S-C, NS versus S-NS, siRNA-*Ldhc* versus S-siRNA-*Ldhc*) (*P*<0.01), while there was no significant difference between the C and NS, S-C and S-NS groups (Fig. 4C). ATP levels were significantly lower in the siRNA-*Ldhc* and S-siRNA-*Ldhc* groups compared with the C and NS groups (*P*<0.01); they were also lower in the S-siRNA-*Ldhc* group compared with the siRNA-*Ldhc* group (*P*<0.01), while there was no significant difference between the C and NS, S-C and S-NS groups (Fig. 4D). In summary, LDH activities, LD content and ATP levels in the siRNA-*Ldhc* group were reduced by 28.21%, 48.38% and 27.88%, respectively, in the skeletal muscle compared with the C group (Fig. 4E).

## DISCUSSION

LDH-C_4_ is one of the members of the lactate dehydrogenase family, which catalyses the terminal reaction in the glycolytic pathway (Blanco and Zinkham, 1963; Goldberg, 1963). LDH-C_4_ displays a unique structure and functional properties (Blanco and Zinkham, 1963; Goldberg, 1963). In other species, the enzymatic kinetic properties of LDH-C_4_ have been studied in detail. Compared with murine LDH-A_4_ and LDH-B_4_, LDH-C_4_ has a lower *Km* for pyruvate (∼0.030 mmol/l) and a higher *Km* for lactate (∼2.0 mmol/l) (Clausen and Ovlisen, 1965; Wilkinson and Withycombe, 1965; LeVan and Goldberg, 1991; Wong et al., 1997). These findings imply that LDH-C_4_ has an affinity for pyruvate that is 60-fold higher than that for lactate, and suggest that pyruvate turnover to lactate may be high even at high concentrations of endogenous or extracellular lactate (Clausen and Ovlisen, 1965; Wilkinson and Withycombe, 1965; LeVan and Goldberg, 1991; Wong et al., 1997). We also found that pika LDH-C_4_ had a lower *K*m for pyruvate (∼0.052 mmol/l) and a higher *K*m for lactate (∼4.934 mmol/l), and that it was beneficial to catalyse the conversion of pyruvate to lactate even at high concentrations of lactate endogenously (Wang et al., 2016). Those results suggest that the biochemical properties, which separated LDH-C_4_ from the other LDH isoforms are conducive to the high glycolytic flux level. The theory is supported by results that ATP production in capacitating spermatozoa could not be affected by an increase in excess lactate (>50-fold for pyruvate) (Hereng et al., 2011). The conversion mediated by LDH could not produce ATP, but it needs NADH as a cofactor to be oxidated to NAD+, and NAD+ concentration is the key factor to limit the glycolysis rate, and is necessary for continued glycolysis (Odet et al., 2011).

Some research has indicated that inhibition of LDH-C_4_, or disruption of *Ldh-c*, in sperm gives rise to a prompt decrease in ATP levels (Coronel et al., 1983; Odet et al., 2008), lower progressive motility, and failure to develop hyperactivated motility. Metabolic tracing results uncovered that all consumed ^13^C was converted to lactate, not oxidized in the tricarboxylic acid cycle. The ATP concentration was >50% in the presence of exogenous pyruvate (Hereng et al., 2011). Compared with suppression without carbonyl cyanide m-chlorophenylhydrazone (CCCP), suppression of oxidative phosphorylation with CCCP and sodium cyanide (NaCN) in mitochondria could result in vigorous sperm motility and maintain the amount of ATP at an equivalent level (Mukai and Okuno, 2004; Hereng et al., 2011). In summary, LDH-C_4_ is the key factor for sperm glycolysis, which has an important role in sperm motility by providing ATP (Coronel et al., 1983; Williams and Ford, 2001; Mukai and Okuno, 2004).

In our previous study, *Ldh-c* was shown to express generally in somatic tissues of plateau pika (Wang et al., 2013). To reveal the physiological mechanisms of LDH-C_4_ in skeletal muscle of plateau pika, we studied the effect of *N*-isopropyl oxamate on pika exercise tolerance (Wang et al., 2015), and found that the swimming times of pikas injected with *N*-isopropyl oxamate in the biceps femoris of the hind legs were significantly lower than those of untreated pikas. LDH activities, LD content and ATP levels in treated pikas were also decreased significantly (Wang et al., 2015).

To further shed light on the role and physiological mechanisms of LDH-C_4_ in skeletal muscle of plateau pika, we investigated the silencing of *Ldh-c* by RNAi on pika exercise tolerance in this study. Our results showed that siRNA-*Ldhc* only interfered with the expression of *Ldh-c* without any effect on *Ldh-a* and *Ldh-b*, implying that the decline in performance tolerance of plateau pika was only due to the silencing of *Ldh-c* in skeletal muscle. The mRNA and protein expression levels in the biceps femoris of the siRNA-*Ldhc* group was decreased by 82.18% and 82.29%, respectively, compared to the control group, while the siRNA-NS group showed no significant difference to the control group. Additionally, LDH isozyme electrophoresis results showed that LDH-C_4_ was downregulated in the siRNA-*Ldhc* group, and LDH activities, LD content and ATP levels in the biceps femoris of siRNA-*Ldhc* pikas were decreased significantly compared to the C group. The swimming time of the siRNA-*Ldhc* group pikas had decreased significantly compared to the C and siRNA-NS group pikas. Therefore, consistent with our previous study (Wang et al., 2015), both using the specific inhibitor of LDH-C_4_ activity and silencing *Ldh-c* to reduce the content of LDH-C_4_ could decrease the level of anaerobic glycolysis in pika skeletal muscle.

In conclusion, pikas enhance their anaerobic glycolysis levels through the expression of *Ldh-c* in skeletal muscle, reducing their dependence on oxygen and increasing their sprint-running capacity in a hypoxia environment.

## MATERIALS AND METHODS

### siRNA plasmids and adenoviral construction

Multiple-site targeting has been proven to have a much stronger RNAi effect (Song et al., 2008). We constructed pMultiRNAi-*Ldhc* to target two sites (codons 321 and 855) of the plateau pika *Ldh-c* gene (accession number HQ704678 in GenBank). The sequences of codons 321 and 855 of *Ldhc* were TTAGTACTTCAAAGATTAC and GGGCTATTGGACTGTCTGTGA, respectively.

To generate pMultiRNAi-*Ldhc*, the pGenesil10-2p plasmid (Wuhan Cell Maker Biotechnology Co. Ltd, Wuhan, China) was used as template DNA using two sets of interfere sequences as primers: GTAATCTTTGAAGTACTAA and TCACAGACAGTCCAATAGCCC. The PCR detection system with cycling conditions was 94°C for 5 min; and then 40 cycles at 94°C for 30 s, 60°C for 30 s and 72°C for 5 min, finishing with chain extension at 72°C for 10 min. The two PCR fragments were linearized with *BsaI* and purified with a Qiagen gel extraction kit (Qiagen, Hilden, Germany) prior to *in vitro* transcription, and were ligated with the T_4_ ligation enzyme. The ligation product was transformed into *E.coli* DH-5α and grown in medium with Kan^+^ to produce pMultiRNAi-*Ldhc*. An RNAi vector targeting a nonspecific sequence (pMultiRNAi-NS) was generated as a control. Similarly, pMultiRNAi-NS was generated using two sets of primers: AACCACATCGCTACTACGACT and GGTGCTCTTCATCTTGTTG.

The pMultiRNAi-*Ldhc* and pMultiRNAi-NS were cloned into adenoviral plasmids (ViraPower Adenoviral Expression System, Invitrogen, USA) with LR homologous recombination *in vitro* and then transfected into the HEK 293 complementation cell line (ATCC, Manassas, USA). After virus propagation and purification, infectious units (iu) were titrated.

### Animal procedure

Plateau pikas were live-trapped at Laji Mountain in Guide County, Qinghai Province, China, at an altitude of 3850 m from 08:00 to 10:00 on 1 October, 2014. To avoid injury to plateau pikas, we handmade the clasps with string and put the clasp over the pikas' cave entrance and fixed it. The pikas were trapped when they passed in and out. To ensure the experimental animals were adults and in good health, the pikas that had body weight <150 g (the body weight of a juvenile pika is <150 g), dishevelled and dull hair, or bradykinesia, were discarded. The average body weight of the plateau pikas was 198±9 g. They were randomly divided into six groups (sample size was eight for each group) and treated as follows. Group 1 (control group, C): 0.5 ml normal saline was injected into each bilateral biceps femoris of the hind legs; Group 2 (swimming control group, S-C): 0.5 ml normal saline was injected into each bilateral biceps femoris of the hind legs and the pikas were forced to swim until exhausted; Group 3 (nonspecific sequence group, NS): 4×10^9^ PFU adenovirus pMultiRNAi-NS was injected into each bilateral biceps femoris of the hind legs; Group 4 (swimming nonspecific sequence group, S-NS): 4×10^9^ PFU adenovirus pMultiRNAi-NS was injected into each bilateral biceps femoris of the hind legs and the pikas were forced to swim until exhausted; Group 5 (siRNA-*Ldhc* group): 4×10^9^ PFU adenovirus pMultiRNAi-*Ldhc* was injected into each bilateral biceps femoris of the hind legs; Group 6 (swimming siRNA-*Ldhc* group, S-siRNA-*Ldhc*): 4×10^9^ PFU adenovirus pMultiRNAi-*Ldhc* was injected into each bilateral biceps femoris of the hind legs and the pikas were forced to swim until exhausted. The point at which animals lost their ability to take the initiative to swim was determined by the condition that they were about to sink below the water. All animals were raised in a trapped site for 5 days after injection of the adenovirus or normal saline before the swimming experiment. After the experiment, all animals were anaesthetized with sodium pentobarbital (5%) and then sacrificed by cervical dislocation immediately before dissection. Skeletal muscle samples were rapidly frozen in liquid nitrogen for long-term storage. All procedures involved in the handling and care of animals were in accordance with the China Practice for the Care and Use of Laboratory Animals and were approved by the China Zoological Society (permit number GB 14923-2010).

### Exercise tolerance experiments on plateau pikas

During long-term evolution, to escape predation effectively, the plateau pika became a sprint-running animal. We found through experimental observation that plateau pikas cannot continuously sprint in the artificial equipment compared to in its indigenous habitats; however, plateau pikas can sprint-swim in water. Therefore, to measure the effect of LDH-C_4_ on exercise tolerance of pikas, forced swimming tests were performed on the S-C, S-NS and swimming siRNA-*Ldhc* groups. The swimming pool was a round water channel with a 30 cm diameter length and 50 cm depth. The ambient temperature in the trapped site was 10-11°C from 08:00 to 10:00 on 1 October, 2014. We therefore controlled the water temperature at 9-10°C. The sample size was eight for each group. In the three groups, the swimming times were recorded in each pika by the maximum seconds they could survive in water.

### RNA extraction and quantification of Ldh-c mRNA level by real-time PCR

Total RNA was isolated with TRIzol reagent (Invitrogen, Carlsbad, USA). The RNA concentration and purity were checked with UV spectrophotometry (1.8 <A260/A280<2.0). RNA integrity was assessed using electrophoresis. A reverse transcription reaction was carried out starting with 4 μg total RNA using the First Strand cDNA Synthesis kit (Thermo Fisher Scientific, Boston, USA). To construct standard curves, 1 μl cDNA was amplified with the Premix Ex Taq Version Kit (TaKaRa Bio Inc, Kusatsu, Japan), and quantification of PCR products were used for plotting standard curves. The initial product concentration was set at 1 and standard curves were generated using a 10-fold serial dilution ranging from 1 to 10^−7^.

Real-time PCR was performed using the SYBR Premix Ex Taq™ II Kit (TaKaRa Bio Inc) protocol on a CFX Connect Real-Time PCR Detection System (Bio-Rad, Hercules, USA.) with cycling conditions of 95°C for 3 min, and then 40 cycles at 95°C for 30 s and 60°C for 30 s. *β-actin* was used as an internal control (Radonić et al., 2004; Li et al., 2009). The PCR special primers for *Ldh-a*, *Ldh-b*, *Ldh-c* and *β-actin* were designed as follows: *Ldh-a*-*F*, 5′-TTGGTCCAGCGGAATGTA-3′, *Ldh-a*-R, 5′-GGTGAACTCCCAGCCTTT-3′, with an amplicon length of 220 bp; *Ldh-b*-F, 5′-TGTTGGACAAGTCGGAATG-3′, *Ldh-b*-R, 5′-CTGAAGAAACAGGCTCCC-3′, with an amplicon length of 139 bp; *Ldh-c*-F, 5′-TATCGAGAATCTGATCGCAGAAGAC-3′, *Ldh-c*-R, 5′-GGGCAAGTTCATCAGCCAAATCC-3′, with an amplicon length of 130 bp. *β-actin*-F, 5′-CTCTTCCAGCCCTCCTTCTT-3′, *β-actin*-R, 5′-AGGTCCTTACGGATCTCCAC-3′, with an amplicon length of 98 bp. The *Ldh-c* mRNA expression level was normalized with *β-actin* to compensate for variations in initial RNA amounts. The relative concentrations of *Ldh-c* and *β-actin* were directly generated by the standard curves, dividing the logarithmic value of *Ldh-c* by the logarithmic value of *β-actin* to obtain normalized expression values.

### Western blot analysis

Total cellular proteins were homogenized and lysed in RIPA Lysis Buffer (Pierce Biotechnology, Rockford, USA) containing protein inhibitors. Protein concentration was measured using a BCA protein assay kit (Pierce Biotechnology). Proteins were separated by sodium dodecyl sulfate polyacrylamide gel electrophoresis (SDS-PAGE) and transferred onto a 0.22 mm polyvinylidene difluoride (PVDF) membrane. After blocking the nonspecific binding sites with 5% nonfat milk for 2 h, the membranes were incubated with a rabbit monoclonal antibody against LDHC (Sigma-Aldrich, Saint Louis, USA; 1:4000 dilution) or GAPDH (Genetex, San Antonio, USA; 1:5000 dilution) at 4°C overnight. The membranes were then washed with TBST (Tris-Buffered Saline with Tween-20) six times at room temperature for 10 min. After washing, the target protein was probed with horseradish peroxidase (HRP)-conjugated goat anti-rabbit IgG antibody (Santa Cruz Biotechnology, Santa Cruz, USA; 1:6000 dilution) at 37°C for 2 h. After washing 10 times with TBST, the bound antibody was checked by chemiluminescence with the ECL Detection Reagent (Pierce Biotechnology).

### LDH activities, LD content and ATP level assay

The skeletal muscle samples of pikas were homogenized on ice as a 1:9 (*W/V*) dilution in 0.9% normal saline. The supernatant was collected after the homogenate was centrifuged at 15,000 r/min at 4°C for 10 min. The LDH activities and LD contents were determined using commercially available assay kits according to the manufacturer's instructions (Nanjing Jiancheng Bioengineering Institute, Nanjing, China). The reaction conditions for the LDH activity assay were measured at 37°C, and absorbance was assessed at 440 nm. The amount of ATP was measured by the luciferin-luciferase method (John, 1970) according to the protocols of the ATP detection kit (Beyotime, Shanghai, China). The luminescence from a 100 μl sample was measured with a luminometer (GloMax 20/20, Promega, Madison, USA) together with 100 μl ATP detection buffer from the ATP detection kit. Standard curves were also generated and the protein concentration of each sample was measured using a BCA Protein assay kit (Pierce Biotechnology).

### LDH isozymes electrophoresis

The skeletal muscle samples of pikas were homogenized on ice as a 1:4 (*W/V*) dilution in 0.9% physiological saline. The homogenate was centrifuged at 15,000 r/min at 4°C for 10 min and the supernatant collected. LDH isozymes electrophoresis was performed with a DY-200 steady current and voltage electrophoresis apparatus (Beijing Liuyi Instrument Factory, Beijing, China). The electrode buffer was Tris-glycine (pH 8.3), and 6 μl of the samples were loaded. The current was 10 mA in the stacking gel and then 25 mA in the separating gel. The LDH bands were stained in the dark at 37°C in a mixture of 4 ml of 5 mg/ml NAD^+^, 2.5 ml of 0.1 mol NaCl, 10 ml of 1 mg/ml nitrobenzene thiocyanate chloride (NBT), 1 ml of 1 mg/ml phenazine methosulfate (PMS), 2.5 ml of 1 mol/l sodium lactate and 0.5 mol/l phosphate buffer (pH 7.5) for 30 min. After rinsing the gels with distilled water, they were stored in 10% glycerol and 7% acetic acid.

### Data analysis

All values were expressed as mean±standard deviation (s.d.). Statistical analysis was performed using SPSS 17.0 software (SPSS Inc, Chicago, USA) by a one-way analysis of variance (ANOVA) and Duncan's test. Before performing an ANOVA, a Kolmogoroe-Simirnov and Levene test was used to detect for normality and homogeneity of variance. *P*<0.05 was considered statistically significant.
